# Caspase-3 promotes oncogene-induced malignant transformation via EndoG-dependent Src-STAT3 phosphorylation

**DOI:** 10.1038/s41419-024-06884-3

**Published:** 2024-07-09

**Authors:** Chenchen Zhu, Fushun Fan, Chuan-Yuan Li, Yan Xiong, Xinjian Liu

**Affiliations:** 1 Department of Biochemistry, School of Medicine, Shenzhen Campus of Sun Yat-sen University, Shenzhen, Guangdong China; 2 BeBetter Med Inc., Guangzhou, Guangdong China; 3 Department of Dermatology, Duke University Medical Center, Durham, NC USA; 4 Guangzhou Consen Pharmaceutical Technology Co. Ltd, Guangzhou, Guangdong China

**Keywords:** Oncogenes, Cell death

## Abstract

Accumulating evidence suggests that caspase-3 plays critical roles beyond apoptosis, serving pro-survival functions in malignant transformation and tumorigenesis. However, the mechanism of non-apoptotic action of caspase-3 in oncogenic transformation remains unclear. In the present study, we show that caspase-3 is consistently activated in malignant transformation induced by exogenous expression of oncogenic cocktail (c-Myc, p53DD, Oct-4, and H-Ras) in vitro as well as in the mouse mammary tumor virus-polyomavirus middle T antigen (MMTV-PyMT) mouse model of breast cancer. Genetic ablation of *caspase-3* significantly attenuated oncogene-induced transformation of mammalian cells and delayed breast cancer progression in MMTV-PyMT transgenic mice. Mechanistically, active caspase-3 triggers the translocation of endonuclease G (EndoG) from mitochondria, which migrates to the nucleus, thereby induces phosphorylation of Src-STAT3 signaling pathway to facilitate oncogenic transformation. Taken together, our data suggest that caspase-3 plays pivotal role in facilitating rather than suppressing oncogene-induced malignant transformation of mammalian cells.

## Introduction

Activation of caspases to induce apoptosis has been the prevailing concept in many cytotoxic chemotherapy and ionizing radiation treatments for cancer [1–3]. However, there is growing recognition of the critical roles that caspases play beyond apoptosis, extending to cellular differentiation, dedifferentiation, tumorigenesis and carcinogenesis [4–6]. For instance, caspases are known contributors to the differentiation of various cell types, including hematopoietic stem cells [7], osteoclasts [8], embryonic stem cells [9, 10], and induced pluripotent stem cells [11]. Moreover, accumulating data indicate that caspases are involved in tumorigenesis by contributing to tumor regrowth [12, 13], therapeutic resistance [14, 15] and angiogenesis [16, 17]. Specifically, activated caspase-3 in dying tumor cells regulates its downstream growth-stimulating signal prostaglandin E2 (PGE2), stimulating the repopulation of surviving tumor cells undergoing radiotherapy [12]. In near-death cancer cells post chemotherapy, active caspase-3 facilitates chemotherapy-induced cancer metastasis [18]. Notably, in human subjects with cancer, higher levels of activated caspase-3 in tumor tissues are correlated with significantly increased rates of recurrence and death [12, 19].

Contrary to their perceived roles as tumor suppressors, sub-lethal activation of the executioner caspases promotes genetic instability and carcinogenesis induced by chemicals, radiation, and oncogene such as Myc [20]. Caspase-3 deficiency has been associated with significantly reduced radiation-induced chromosome aberrations and DMBA (7,12-Dimethylbez[a]anthracene)/TPA(12-OTetradecanoylphobol-13-acetate)-induced skin carcinogenesis in transgenic mice [19]. These studies counterintuitively demonstrated that caspase-3 plays a double-edged role in carcinogenesis and tumorigenesis. While the counterintuitive roles of caspase-3 as a pro-oncogene in chemical-, radiation-induced carcinogenesis, and chemo-, radiotherapy-induced tumorigenesis have been largely established, the relationship between the non-apoptotic actions of caspase-3 in oncogene-induced transformation are not completely understood.

In the current study, we attempted to elucidate the non-apoptotic role of caspase-3 in oncogene-induced transformation using exogenous expression of oncogenic cocktail (c-Myc, p53DD, Oct-4, and H-Ras) in vitro and in MMTV-PyMT transgenic mouse model. Our findings demonstrate that caspase-3 activation prompts the translocation of its downstream effector EndoG into the nucleus, activating Src and STAT3 phosphorylation to promote oncogene-induced malignant transformation.

## Results

### Activation of caspase-3 during the oncogene-mediated transformation process

**Fig. 1:**
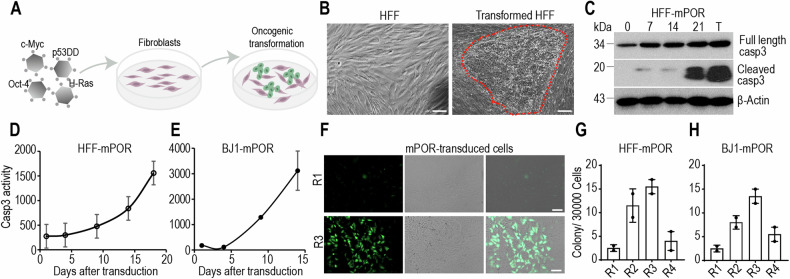
Caspase-3 activation in oncogene-induced transformation. **A** Schematic representation of normal fibroblasts reprogrammed into malignant cells by exogenous expression of oncogenic cocktail (c-Myc, p53DD, Oct-4, and H-Ras, mPOR). **B** Representative transformed colony of mPOR-transduced HFF cells at day 21 post transduction. The red demarcated line indicates the transformed colony. The scale bar represents 200 μm. **C** Western blot analysis of cleaved caspase-3 in HFF cells at different time points post mPOR oncogene transduction. T represents malignant transformed cells. β-Actin was used as protein loading control. The blots for cleaved-casp3, full-length casp3 and β-Actin were run on separate gels. The dynamic luciferase activity was monitored in Casp3-Luc-GFP stably expressing HFF (**D**) and BJ1 (**E**) cells at different time points post mPOR transduction. Data are presented as mean ± SD, *n* = 3. **F** GFP fluorescence showing low (R1) and high (R3) level of caspase-3 activation in the colonies derived from mPOR-transduced Casp3-Luc-GFP stably expressing HFF cells. Scale bar represents 200 μm. Morphologically transformed colonies outgrowth in four subpopulations (R1-R4) of Casp3-Luc-GFP stably expressing HFF (**G**) and BJ1 (**H**) cells after mPOR transduction. Data are presented as mean ± SD, *n* = 2.

To induce fibroblasts transformation into malignant cancer cells, we used an established protocol to generate of cancer stem-like cells from primary human cells through the expression of defined genetic factors [21]. Combined transduction of four oncogenic factors c-Myc, p53DD, Oct-4, and H-Ras (mPOR)-induced efficient transformation of human fibroblasts into malignant cells with loss of contact inhibition and gaining the ability to form dense colonies (Fig. 1A, B).

In order to monitor caspase-3 activity in the process of oncogene-mediated transformation, mPOR-transduced human fibroblasts were harvested in every week. Western blot analysis shows that active caspase-3 activity in mPOR-transduced cells was progressively increased in a time-dependent manner, with the highest level of caspase-3 activation in transformed colony (Fig. 1C).

We further used a noninvasive caspase-3 reporter which consist of a firefly luciferase-GFP fusion protein (Luc-GFP) linked to a polyubiquitin domain to monitor caspase-3 activity in the process of transformation (Fig. S1A). Caspase-3 Luc-GFP reporter stably expressing fibroblasts were transduced with mPOR factors to initiate the transformation process. Oncogene cocktail induced significant caspase-3 reporter activation in a persistent manner (Fig. 1D, E). These oncogene-transduced cells tolerated caspase-3 activation to survive and proliferate, which were consistent with the radiation-induced caspase-3 activation [19]. The mPOR-transduced caspase-3 reporter fibroblasts were sorted to four sub-populations (R1-R4) by use of FACS according to the cellular fluorescence intensities on day 10. They were, then re-plated to evaluate their colony formation ability of sub-population with different level of caspase-3 activity (Fig. S1B). It appeared that cells could tolerate a wide range of caspase-3 activation levels (Fig. S1B). In colonies that eventually emerge from mPOR-transduced cells, most (about 80%) of the colonies were positive for caspase-3 reporter when observe through GFP fluorescence (Fig. 1F). Interestingly, those with relative higher caspase-3 activities (Fig. 1G, H**: R2, R3** in both cell lines) formed colonies at significantly greater frequencies than those with low caspase-3 activities, excluding instances of the highest level of caspase-3 leading to apoptotic cell death (Fig. 1G, H**: R1** in both cell lines). Those data suggest that relative higher caspase-3 activities correlated with significantly higher frequencies of oncogenic transformation.

### Caspase-3 promotes oncogenic transformation

**Fig. 2:**
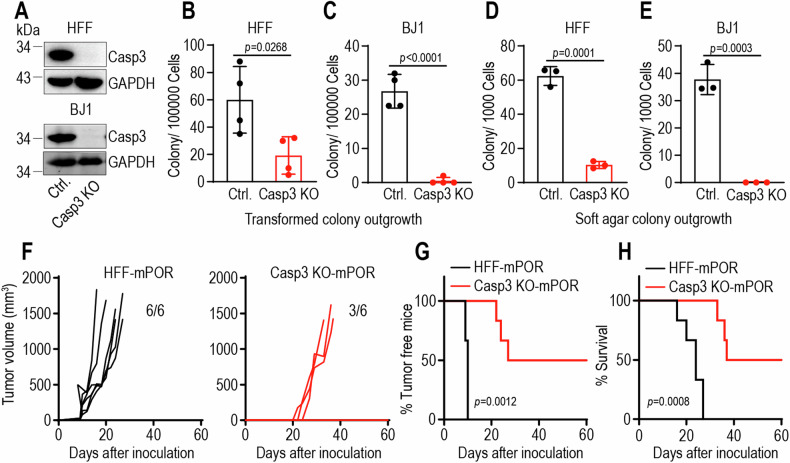
Caspase-3 deficiency attenuates oncogene-induced malignant transformation. **A** Caspase-3 expression in control (Ctrl.) and caspase-3 knockout (Casp3 KO) HFF (upper panel) and BJ1 cells (lower panel). GAPDH was used as protein loading control. The blots for Casp3 and GAPDH were run on the same gel for each cell lines, with blots for BJ1 cells being duplicated. Morphologically transformed colony outgrowth in mPOR-transduced control and Casp3 KO HFF (**B**) and BJ1 cells (**C**). Data are presented as mean ± SD, *n* = 4. *p* values were determined using Student’s *t*-test. Soft agar colony growth from transformed control and Casp3 KO HFF (**D**) and BJ1 (**E**) cells. Data are presented as mean ± SD, *n* = 3. *p* values were determined using Student’s *t*-test. **F** Tumor growth curve of 1 × 10^6^ mPOR-transduced HFF cells (HFF-mPOR) and Casp3 KO cells (Casp3 KO-mPOR) subcutaneous injection into female BALB/c nude mice. *n* = 6 per group. 3 tumor-free mice in Casp3 KO-mPOR group till on day 60. **G** Kaplan–Meier plot showing the percentage of tumor-free mice in HFF-mPOR and Casp3 KO-mPOR groups. *p* values were determined by Log-rank *t*-test. **H** Kaplan–Meier plot showing the survival rate of HFF-mPOR and Casp3 KO-mPOR groups. *p* values were determined by Log-rank *t*-test.

We further examined if a causative relationship exists between caspase-3 activation and oncogenic transformation by use of CRISPR technology knocking out *caspase-3* gene (Casp3 KO) in fibroblasts (Fig. 2A). Caspase-3 knockout significantly decreased oncogenic transformation rates of mPOR-transduced fibroblasts in vitro models (Fig. 2B, C). The ability to grow in an anchorage-independent manner in soft agar is a hallmark of transformed cells [22]. Our data show that caspase-3 knockout significantly decreased the soft agar formation ability of mPOR-transduced cells (Fig. 2D, E). Our results suggest that a significantly facilitative role of caspase-3 in oncogene-mediated transformation. We further confirmed the tumorigenic nature of the mPOR-transduced control and Casp3 KO cells in xenograft mouse model. In contrast to the robust tumor-forming capabilities observed in mPOR-transduced control cells, putative transformed cells with caspase-3 deficiency exhibited significantly delayed tumor formation (Fig. 2F, G), with prolonged lifespan of tumor-burdened mice (Fig. 2H). The above experiments clearly demonstrate that caspase-3 facilitates oncogenic transformation of human fibroblasts, especially for in vivo tumor formation.

### Caspase-3 depletion delays breast cancer progression in MMTV-PyMT transgenic mice

MMTV-PyMT transgenic mice express the Polyoma Virus Middle T antigen under the direction of the mouse mammary tumor virus promoter/enhancer, leading to the development of palpable mammary tumors in females. This genetically engineered mouse mode is widely utilized for studying oncogene-induced transformation.

**Fig. 3:**
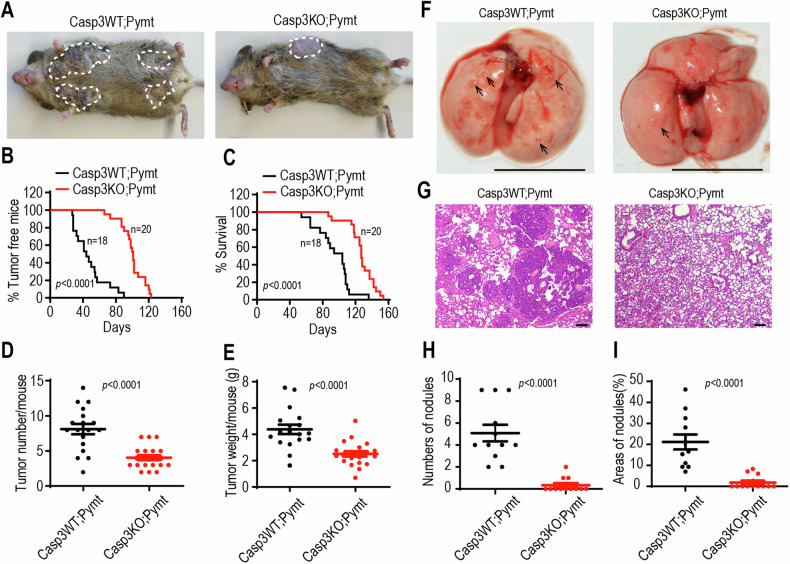
Caspase-3 depletion delays breast cancer progression in MMTV-PyMT transgenic mice. **A** Representative image of wild-type (Casp3WT;Pymt) and Casp3 deficient (Casp3KO;Pymt) virgin female MMTV-PyMT transgenic mice at 13 weeks old, showing grossly visible tumors (demarcated by dotted lines). **B** Kaplan–Meier plot showing the percentage of tumor-free mice in wild type (Casp3WT;Pymt, *n* = 18) and Casp3 deficient (Casp3KO;Pymt) virgin female MMTV-PyMT transgenic mice (*n* = 20). *p* values were determined by Log-rank *t*-test. **C** Kaplan–Meier plot showing the survival rate of wild type (Casp3WT;Pymt) and Casp3 deficient (Casp3KO;Pymt) virgin female MMTV-PyMT transgenic mice. *p* values were determined by Log-rank *t*-test. **D** Number of tumors per mouse in wild type (Casp3WT;Pymt) and Casp3 deficient (Casp3KO;Pymt) MMTV-PyMT transgenic mice at the endpoint. *p* values were determined using Mann-Whitney *U*-test. **E** Weight of tumors per mouse in wild type (Casp3WT;Pymt) and Casp3 deficient (Casp3KO;Pymt) MMTV-PyMT transgenic mice at the endpoint. Data are presented as mean ± SD. *p* values were determined using Student’s *t*-test. **F** Gross photography showing apparent pulmonary metastases in Casp3WT;Pymt mice and Casp3KO;Pymt mice. The black arrow indicates the site of the tumor nodule. The scale bars represent 1 cm. **G** Representative hematoxylin and eosin staining of lung sections from Casp3WT;Pymt and Casp3KO;Pymt mice. The scale bars represent 100 μm. **H** The number of metastatic tumor burden in the random fields of lung sections (*n* = 11 per group). **I** The percentage of tumor area coverage in each random field of lung sections (*n* = 11 per group). Data are presented as mean ± SD in (**H, I**). *p* values were determined using Mann–Whitney *U*-test in (**H, I**).

To confirm the facilitative role of caspase-3 in oncogene-mediated transformation, caspase-3 deficient (Casp3 KO) mice [23] were crossed to MMTV-PyMT transgenic mice predisposed to malignant mammary adenocarcinoma as a result of mammary gland-directed expression of a Pymt oncogene [24]. We followed a prospective cohort consisting of virgin female Casp3 knockout/PyMT positive (Casp3KO;Pymt, *n* = 20) mice and of Casp3 wild type/PyMT positive (Casp3WT;Pymt, *n* = 18) female littermate control mice to determine the impact of caspase-3 deficiency on primary mammary tumor development. Caspase-3 knockout caused a profound reduction in tumorigenesis (Fig. 3A). The median age of development of the first palpable mammary tumor of Casp3KO;Pymt mice was 100 days with a range of 66 to 123 days, as a compared to 47.7 days in Casp3WT;Pymt mice with a range of 27 to 90 days (Fig. 3B). The mice were scarified when any burdened tumor over 1.5 cm in diameter. All tumors in scarified mice were counted and weighted. Our data show that caspase-3 deficiency significantly prolonged the lifespan of mice with tumors (Fig. 3C). Furthermore, Casp3KO;Pymt mice were burdened with significantly less tumor numbers (Fig. 3D) with lighter tumor weight (Fig. 3E) per mouse on the end of experiment. These data indicate that caspase-3 activities are required for tumorigenicity in oncogene-induced breast cancer mouse model. Additionally, the MMTV-PyMT mouse model of breast cancer exhibits an exceptionally aggressive phenotype, with a lung metastatic rate that exceeds 90%. We further examined the impact of caspase-3 on lung metastasis in these MMTV-PyMT mice. Gross images of the Casp3WT;Pymt mice revealed pronounced lung metastases, while Casp3KO;Pymt mice displayed only a limited number of metastatic tumor masses (Fig. 3F). Representative hematoxylin and eosin (H&E) staining further confirmed the restricted presence of metastatic lung tumors in the Casp3KO;Pymt mice (Fig. 3G–I). To gain insight into the potential role of caspase-3 expression in malignant transformation, we utilized the TNMplot database, encompassing 15,648 normal and 40,442 tumor samples [25], to analyze the expression levels of caspase-3. In comparison with normal tissues, caspase-3 exhibited high expression in various cancer types notably breast cancer (Fig. S2A), thereby reinforcing the indication that caspase-3 plays a crucial role in tumorigenesis.

### Endonuclease G as a major downstream effector of caspase-3-mediated transformation

EndoG, an endonuclease associated with DNA fragmentation during apoptosis, typically resides within mitochondria. However, upon exposure to cell death stimuli, EndoG can translocate from mitochondria to the nucleus [26, 27]. To investigate whether active caspase-3 directly instigates the release of EndoG from mitochondria, we extracted mitochondria from mammary cells. The mitochondria (20 μg) were incubated with or without recombinant active human caspase-3 (0.4 μg, BD Biosciences) in caspase-3 assay buffer (20 mM HEPES, pH 7.4, 0.1% CHAPS, 5 mM DTT, 2 mM EDTA) supplemented with protease/phosphatase inhibitors at 37 °C for 1 h or 2 h. The supernatant was then separated from treated mitochondria, and analyzed by western blot using anti-EndoG antibody. As shown in Fig. S3A, S3B, recombinant active caspase-3 induced the release of EndoG from isolated mitochondria. However, whether this direct effect of caspase-3 on mitochondria is enzymatic activity-dependent need to be further investigated.

**Fig. 4:**
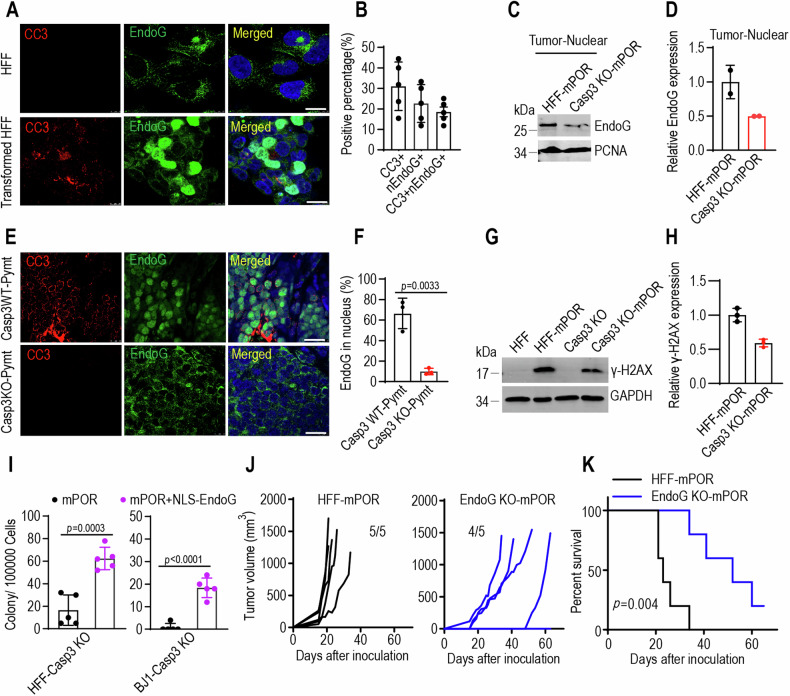
EndoG translocation in oncogene-induced malignant transformation. **A** Immunofluorescence staining image of cleaved caspase-3 (red) and EndoG (green) in parental HFF and mPOR-transduced HFF cells on day 21. The scale bar represents 10 μm. **B** The positive percentage of cleaved caspase-3 (CC3), nuclear EndoG (nEndoG), and double positive (CC3+nEndoG) in mPOR-transduced HFF cells of (**A**). Data from five random photography fields. Western blot images (**C**) and quantification (**D**) showing low expression of EndoG in the nucleus of caspase-3-deficiency mPOR-derived tumor samples. PCNA was used as nuclear protein loading control. The blots for EndoG and PCNA were run on separate gels, with each blot being duplicated. Immunofluorescence staining image (**E**) and quantification (**F**) of cleaved caspase-3 (red) and EndoG (green) in tumor samples from wild type (Casp3WT;Pymt) and Casp3 knockout (Casp3 KO;Pymt) MMTV-PyMT transgenic mice. Scale bar represents 10 μm. Data from three random photography fields. *p* value was determined using Student’s *t*-test. Western blot images (**G**) and quantification (**H**) showing the activation of H2AX (γ-H2AX) in mPOR-transduced fibroblasts and caspase-3 deficiency fibroblasts. GAPDH was used as protein loading control. The blots for γ-H2AX and GAPDH were run on the same gel, with each blot being triplicated. **I** Morphologically transformed colonies outgrowth in caspase-3 deficient mPOR-transduced HFF (left panel), BJ1 (right panel) cells in the presence of NLS-EndoG. Data are presented as mean ± SD, *n* = 5. *p* values were determined using Student’s t-test. **J** Tumor growth curve of 1 × 10^6^ mPOR-transduced HFF cells (HFF-mPOR) and EndoG KO cells (EndoG KO-mPOR) subcutaneous injection into female BALB/c nude mice. *n* = 5 per group. 1 tumor-free mice in EndoG KO-mPOR group till on day 63. **K** Kaplan–Meier plot showing the survival rate of mPOR-transduced control and EndoG KO HFF cells in female nude mice. *p* value was determined by Log-rank *t*-test.

To determine whether EndoG migrates to nucleus in a caspase-3-dependent manner during oncogene-induced transformation, we carried out immunofluorescence staining and western blotting analysis to identify the location of EndoG in mPOR-transduced fibroblasts. Compared to parental fibroblast, there was notable caspase-3 activation and EndoG translocation to nucleus (nEndoG) in mPOR-transduced fibroblasts (Fig. 4A, B, Fig. S3C, D). Caspase-3 activity appears to be a major regulator of EndoG’s nuclear translocation, as evidenced by reduced nuclear EndoG expression in caspase-3-deficiency mPOR-derived tumor samples (Fig. 4C, D). Importantly, tumor samples from PyMT transgenic mice with caspase-3 deficiency exhibited minimal EndoG nuclear translocation (Fig. 4E, F). Given that EndoG is an endonuclease associated with DNA fragmentation, we observed a heightened level of DNA damage (marked by γ-H2AX as an indicator of double-strand DNA damage) in mPOR-transduced fibroblasts compared to caspase-3 deficiency fibroblasts (Fig. 4G, H). Reintroducing a modified EndoG, where the native mitochondrial localization signal was switched to a nuclear localization signal (NLS-EndoG, Fig. S3E), into mPOR-transduced Casp3 KO fibroblasts (Fig. S3F) significantly increased γ-H2AX expression (Figs. S3G, 3H). These findings demonstrate that EndoG can migrate into the nucleus to fragment nuclear DNA in response to caspase-3 activation.

We further explored the role of EndoG in oncogene-induced transformation by introducing NLS-EndoG into Casp3 KO fibroblasts (Figs. S3I and 3J). Remarkably, nucleus-located EndoG successfully restored mPOR-induced oncogenic transformation in caspase-3 deficient cells (Fig. 4I). To corroborate the tumorigenic impact of EndoG in vivo, mPOR-induced fibroblasts with EndoG deletion (Fig. S3K) were transplanted into nude mice. In contrast to the potent tumor-forming abilities of mPOR-transduced control cells, putative transformed cells with EndoG deficiency exhibited significantly attenuated tumor-forming capabilities, with prolonged survival of syngeneic hosts (Fig. 4J, K). These results suggest that the nuclear migration of EndoG is a crucial prerequisite for oncogene-induced transformation.

### Caspase-3 and EndoG promote stemness via the Src-STAT3 pathway facilitating oncogenic transformation

Now that we have established the significance of activated caspase-3 and nuclear translocation of EndoG in oncogenic transformation, the next logical question is to identify the downstream factors responsible for supporting tumorigenicity. Oncogene-cocktail-transduced cells tolerate constitutive caspase-3 activation for survival, and the nuclear translocation of EndoG induces sublethal DNA double-strand breaks [19, 20], resembling the effects of low-dose genotoxin stress. Previous study demonstrated that a low dose of genotoxin etoposide induces sublethal DNA damage, leading to Src kinases activation and promoting cell survival [28]. We hypothesize that constitutive caspase-3/EndoG activation in oncogene cocktail-transduced cell might promote Src kinases activation to promote cellular transformation. Src kinases are regulatory proteins that play key roles in cell differentiation, proliferation, and survival. The kinase activity of Src depends on whether the protein is in the more expanded “open” active conformation or in the more compact “closed” repressed conformation through phosphorylation at various tyrosine residues, including Tyr416 (Y416), and Tyr527 (Y527). Y527 phosphorylation stabilizes a closed conformation, which suppresses kinase activity towards substrates, whereas phosphorylation at Y416 promotes an elevated kinase activity by stabilizing the activation loop in a manner permissive for substrate binding. In its non-phosphorylated state, Src is localized in an autoinhibited conformation, inhibiting its kinase activity [29–33].

**Fig. 5:**
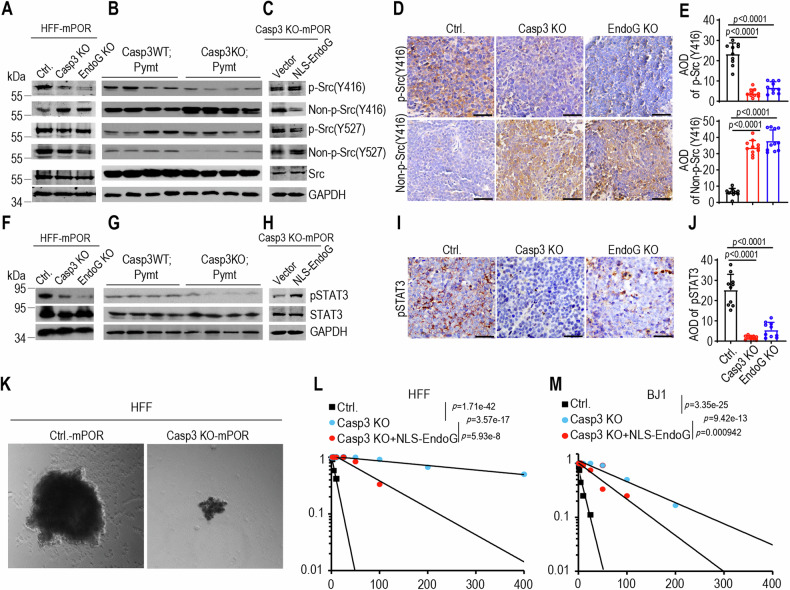
Caspase-3, and EndoG facilitate oncogenic transformation via Src-STAT3 pathway. **A** Western blot analysis of phosphorylation and non-phosphorylation of Src at Y416 and Y527 in control (Ctrl.), Casp3 KO and EndoG KO HFF cells after mPOR oncogene transduction. **B** Western blot analysis of phosphorylation and non-phosphorylation of Src at Y416 and Y527 in tumor samples from wild type (Casp3WT;Pymt) and Casp3 deficient (Casp3KO;Pymt) MMTV-PyMT transgenic mice. Four tumor samples per group. **C** Western blot analysis of phosphorylation and non-phosphorylation of Src at Y416 and Y527 in Casp3 KO-mPOR-transduced fibroblasts in the presence of NLS-EndoG. GAPDH was used as protein loading control for (**A**–**C**). The blots for the target protein and GAPDH were run on separate gels in each experiment (**A**–**C**). IHC staining (**D**) and quantification (**E**) of p-Src(Tyr416) and non-p-Src(Tyr416) in mPOR-transformed HFF control (Ctrl.), Casp3 KO or EndoG KO tumors. AOD = the integrated optical density (IOD)/Area of positive staining in IHC staining image. The scale bars represent 50 μm. Data from ten random photography fields; data are presented as mean ± SD. *p* values were determined using Student’s *t*-test. **F** Western blot analysis of phosphorylation of STAT3 (Y705) in control (Ctrl.), Casp3 KO and EndoG KO HFF cells after mPOR oncogene transduction. The blots for pSTAT3 (Y705) and STAT3 were run on separate gels, while blots for pSTAT3 (Y705) and GAPDH were on the same gel. **G** Western blot analysis of phosphorylation of STAT3 in tumor samples from wild type (Casp3WT;Pymt) and Casp3 deficient (Casp3KO;Pymt) MMTV-PyMT transgenic mice. 4 tumor samples per group. **H** Western blot analysis of phosphorylation of STAT3 in Casp3 KO-mPOR-transduced fibroblasts reintroduced with nuclear-located modified EndoG. GAPDH was used as protein loading control for (**F**–**H**). The blots for the target protein and GAPDH were run on separate gels in each experiment (**F**–**H**), with **H** being duplicated. **I** and **J**, IHC staining (**I**) and quantification (**J**) of pSTAT3 in mPOR-transformed HFF control (Ctrl.), Casp3 KO or EndoG KO tumors. The scale bars represent 50 μm. Data from ten random photography fields; data are presented as mean ± SD. *p* values were determined using Student’s *t*-test. **K** Representative morphology of tumor spheres of mPOR-transduced HFF control and Casp3 KO cells. Extreme limiting dilution assay evaluation of tumor sphere formation abilities of mPOR-transduced HFF (**L**) and BJ1 (**M**) control, as well as Casp3 KO and Casp3 KO with reintroduced NLS-EndoG in both cell lines. *p* value represents chi-square.

Knocking out caspase-3 or EndoG resulted in the downregulation of phosphorylated Tyr416 Src (p-Src Y416) expression, while upregulating the non-phosphorylated Tyr416 Src (p-Src Y416) expression in mPOR-transduced fibroblasts (Fig. 5A). Additional data using tumor samples from Casp3WT;Pymt and Casp3KO;Pymt mice further confirmed the role of caspase-3 in mediating phosphorylation of Src (Figs. 5B and S4A). Introducing NLS-EndoG into caspase-3-deficient or EndoG-deficient transformed cells restored the phosphorylated Tyr416 Src expression, inhibiting non-phosphorylated Tyr416 Src expression (Figs. 5C and S4B). However, caspase-3 or EndoG exerts minimal influence on Tyr527 site, regardless of its phosphorylation or non-phosphorylation state. The causal relationships between caspase-3/EndoG and Src activation was further confirmed through immunohistochemistry staining of tumor samples from caspase-3- or EndoG-deficient xenografts, which indicated the downregulation of phosphorylated Tyr416 Src expression, and upregulation the non-phosphorylated Tyr416 Src expression in caspase-3- or EndoG-deficient tumors (Fig. 5D, E). Introducing a Src kinase inactivation version by transducing the K298M-Y419F double mutant, lacking Y419 (analogous to chicken Y416 [34]) phosphorylation active site and kinase-dead (K298M) (Fig. S4C), inhibited cellular proliferation of HFF-mPOR with no discernible impact on HFF Casp3 KO-mPOR cells (Fig. S4D–F). Conversely, elevated expression of Src protein notably enhanced the proliferation of HFF Casp3 KO-mPOR cells (Fig. S4F). These results demonstrate that oncogene-induced constitutively caspase-3/EndoG/Src activation plays critical role in malignant transformation.

The exogenous expression of the oncogene cocktail induced a significant number of DNA double-strand breaks in human fibroblasts [35]. Within cancer cells, spontaneously sublethal activation of apoptotic caspases and nucleases, resulting in constitutive DNA double-strand breaks and subsequent persistent activation of DNA damage response kinase ATM. Activated ATM, in turn, triggers the activation of transcription factors NF-κB and signal transducer and activator of transcription 3 (STAT3) [36]. STAT3 is an important transcription factor on both tumor growth and maintenance of stemness of cancer cell [36–39]. In our subsequent investigation, we explore the relationship between constitutive caspase-3/EndoG/Src activation and the status of STAT3 in oncogene-induced malignant transformation. Western blot analysis indicated that caspase-3/EndoG knockout significantly reduced the level of phosphorylated STAT3 at Y705 (pSTAT3) in mPOR-transformed cells (Fig. 5F) as well as in tumor samples from Casp3WT;Pymt and Casp3KO;Pymt mice (Fig. 5G). Introducing NLS-EndoG into caspase-3-deficient transformed cells restored the phosphorylated STAT3 expression (Fig. 5H). Immunohistochemistry staining of tumor samples from caspase-3- or EndoG-deficient xenografts further confirmed the relationship between caspase-3/EndoG and pSTAT3 expression (Fig. 5I, J), which is consistent to the phosphorylation of Src at Tyr416.

We further investigated the relationship between Src activation and the phosphorylation of STAT3 in Src constitutively expressing cells. In line with previous study that STAT3 is constitutively activated in Src oncoprotein-induced cell transformation [40, 41], Src transduction led to increased phosphorylation of STAT3, while caspase-3 knockout significantly reduced the level of pSTAT3 in Src-transduced cells (Fig. S4G). Upon deactivating Src activity phosphorylated STAT3 (pSTAT3) maintained at low levels in both wild-type and Casp3 KO cells (Fig. S4G). Previous study demonstrated that the knockout of caspases, nucleases and ATM significantly deceases the phosphorylation of STAT3, the expression of stem cell marker, and reduces the ability of tumor spheres formation [36, 42]. STAT3 is an important transcription factor involved in both tumor growth and maintenance the stemness of cancer cell, as well as embryonic stem cells. Our western blot analysis revealed a remarkable downregulation of p-SRC (Tyr416) and pSTAT3 expression in Casp3 KO and EndoG KO cells (Fig. 5A, F) and tumors from Casp3KO;Pymt mice (Fig. 5B, G). Elevated Src kinase activity has consistently been linked to increased stemness in various cancers [43–45]. We hypothesize that caspase-3 activation facilitates oncogene-induced transformation by modulating the Src-STAT3 signaling pathway, which in turn influences cell stemness. Tumor sphere formation in suspension culture is an important characteristic of cancer stem cells [46]. We conducted the tumor sphere formation assay and our results demonstrated that mPOR-transduced Casp3 KO HFF cells exhibited smaller tumor spheres than mPOR-transduced HFF cells (Fig. 5K). The limiting dilution assay data further validate the fewer tumor sphere numbers of mPOR-transduced Casp3 KO HFF cells compared to mPOR-transduced HFF cells. Re-introduced NLS-EndoG restored the tumor sphere formation ability of mPOR-induced Casp3 KO cells (Fig. 5L, M). These results establish a clear causal relationship between caspase-3/EndoG and cancer stemness thus provide the strong evidence for the role of caspase-3 in driving the tumorigenicity and stemness of oncogene-induced transformation.

## Discussion

Caspase-3 has been well established to function as executioner during apoptotic cell death. In this study, we demonstrated that caspase-3 paradoxically facilitates malignant transformation induced by exogenous expression of oncogenic cocktail in vitro as well as in MMTV-PyMT mouse model of breast cancer. These results are striking and challenge the notion that caspase activation is always anti-tumorigenic.

Although counterintuitive at first glance, our findings are consistent with and may shed further insights into some significant observations made in the process of dedifferentiation and malignant transformation. Previous studies have uncovered that caspase-3 activation reverse the process of cellular differentiation, and promote nuclear reprogramming in the induction of pluripotent stem cells generation [11], embryonic stem cell differentiation [10], and proliferation of stem cells in skin wound healing and liver regeneration after partial hepatectomy [47]. Caspase-3-deficient mice exhibit reduced expression of genes involved in cell cycle progression, leading to abnormal myocardial differentiation [48]. Our previous study demonstrated that caspases activation leads to the persistence of spontaneous DBS in cancer cells, which then activates NF-κB and STAT3 to maintain or enhance tumorigenicity and cancer cell stemness [36]. The impact of caspase-3 activation in maintaining the stemness of both normal cells and cancer cells aligns with our current study, wherein caspases activation induces EndoG nuclear translocation, resulting in DNA damage in transforming cells. This, in turn, activates Src and STAT3, two well-known factors in maintaining the tumorigenicity and stemness of cancer cells [49, 50], propelling malignant transformation.

Sub-lethal levels of caspase activation induced by extrinsic stress and intrinsic apoptotic stimuli can trigger limited mitochondrial outer membrane permeabilization (MOMP). This cascade results in the release of various proteins, including endonucleases such as caspase-activated DNase (CAD) and endonuclease G (EndoG), capable of causing double-stranded DNA cleavage, telomerase reverse transcriptase splicing and genome instability. These continual endogenous DNA damage resulting from failed apoptosis promotes malignant transformation and tumorigenesis [19, 36, 51]. Following DNA damage, early changes in expressed proteins are known to drive cell fate by shifting the balance between pro-apoptotic and pro-survival programs. Src is a non-receptor tyrosine kinase involved in intercellular signaling pathways, and activation of Src plays a role in various cellular processes, including cell proliferation, survival, migration, and differentiation. Src was activated by low but not high levels of DNA damage in genotoxin etoposide-treated human fibroblasts. With low-level DNA damage, Src-mediated activation of p38 critically promoted expression of cell survival proteins, while Src-mediated repression of p53 prevented a rise in proapoptotic proteins. With high-level DNA damage, failure to activate Src led to elevation of p53, inhibition of p38, and apoptosis [28]. In this study, we demonstrated that nuclear translocation of EndoG causes DNA damage and phosphorylation of Src on Tyr416, contributing to oncogenic transformation and cancer stemness. While the impact of DNA damage induced by failed apoptosis on cancer progression is evident, further investigation is needed to elucidate the molecular regulation of Src in this oncogenic transformation process.

**Fig. 6:**
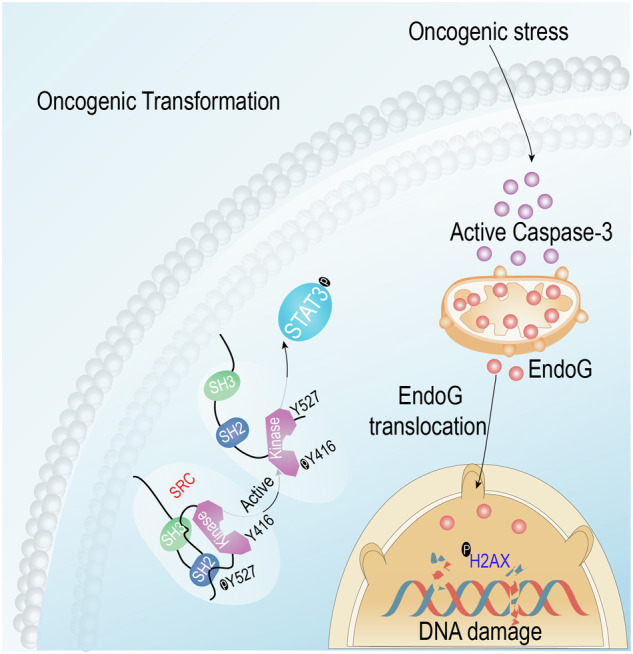
Diagrammatical image illustrates the facilitating role of caspase-3 in oncogenic transformation. Oncogene stress-induced caspase-3 activation triggers the translocation of EndoG from mitochondria, which migrates to the nucleus, thereby induces phosphorylation of Src-STAT3 signaling pathway to facilitate oncogenic transformation.

Taken together, the present study reveals an unexpected function for caspase-3 in oncogene-induced transformation. Contrary to its traditional role in apoptosis, oncogene stress-induced caspase-3 activation triggers the translocation of EndoG from mitochondria, which migrates to the nucleus, thereby induces phosphorylation of Src-STAT3 signaling pathway to facilitate oncogenic transformation (Fig. 6).

## Material and methods

### Cell culture

Early passage, hTERT immortalized non-transformed human foreskin fibroblast cell line, HFF was a kind gift from Dr. Michael Kastan of Duke University (Durham, NC). Human fibroblast BJ1 was obtained from Guangzhou Cellcook Biotech Co. Ltd. The culture medium consisted of DMEM supplemented with 10% fetal bovine serum (Sigma, St. Louis, MO, USA) and 100 units/ml penicillin and 100 μg/ml streptomycin. The cells were tested periodically to ensure the absence of mycoplasma infection.

### CRISPR/Cas9-mediated gene knockout

Caspase-3 and EndoG knockout cell lines were generated by use of lentivirus-mediated CRISPR/Cas9 technology. Single guided RNA (sgRNA) sequences targeting caspase-3 and EndoG genes were designed with the use of a free online CRISPR design tool (crispr.mit.edu). Annealed double-stranded sgRNA oligos were ligated into the BsmB1 locus of lentiCRISPRv2 (deposited by Dr. Feng Zhang of MIT to Addgene, Cambridge, MA), which co-expresses Cas9 and sgRNA in the same vector. The sgRNA-encoding CRISPR lentivirus vector plasmids with the second-generation packaging plasmids (psPAX2, pMD2.G; both deposited at Addgene by Dr. Didier Trono of EPFL (Ecole Polytechnique Fédérale de Lausanne, Switzerland) following a published, calcium phosphate-based Trono Lab protocol (http://tronolab.epfl.ch/page58122.html) were transduced into 293T cells. After 48 h of transfection, supernatants containing lentiviral particles were collected.

Single-guided RNA (sgRNA) sequences used to construct the recombinant vector are as follows:

Caspase3-F-1 5′-CACCGcatacatggaagcgaatcaa-3′;

Caspase3-R-1 5′-AAACttgattcgcttccatgtatgC-3′;

Caspase3-F-2 5′- CACCGggaagcgaatcaatggactc-3′;

Caspase3-R-2 5′-AAACgagtccattgattcgcttccC-3′;

EndoG-F-1 5′-CACCGgggctgggtgcggtcgtcga-3′;

EndoG-R-1 5′-AAACtcgacgaccgcacccagcccC-3′;

EndoG-F-2 5′-CACCGcgacttccgcgaggacgact-3′;

EndoG-R-2 5′-AAACagtcgtcctcgcggaagtcgC-3′.

### Oncogene-induced transformation

The plasmids encoding MycT58A, p53DD, HRasG12V were obtained from Addgene. They were deposited by Dr. Christopher Counter of Duke University (Durham, NC). The plasmid encoding the human OCT4 gene was also obtained from Addgene. It was deposited by Dr. James A. Thomson of University of Wisconsin (Madison, Wisconsin). All of the above genes were transferred into the pLEX lentiviral vector with a human influenza hemagglutinin (HA) tag (Open Biosystems, Huntsville, AL) by use of PCR-mediated subcloning. The lentivirus particles encoding the above genes were packaged following the method described herein for lentivirus-mediated CRISPR/Cas9 technology. Four factors (mPOR; MycT58A, p53DD, Oct-4, HRasG12V) were transduced into fibroblasts to reprogram normal fibroblasts into malignant cancer colonies.

### Molecular cloning

The pLEX lentiviral vectors system was used to deliver reporter and other genes into target cells. A caspase-3 recognition site (DEVD) was engineered into the 5’ end of the luciferase-GFP fusion gene. In addition, a flexible linker (Gly3Ser)3 sequence was incorporated in between the luciferase-GFP fusion gene. The stop codon for the luciferase gene was removed to allow a full read-through. Subsequently, a 9-unit polyubiquitin domain was then ligated into the 5’ end of the luciferase-GFP gene. The fully assembled caspase-3 reporter genes were transferred into the lentiviral vector pLEX (Open Biosystems, Huntsville, AL) monitoring caspase-3 reporter activation and sorting of cells with high or low reporter activities.

Nucleus-targeted EndoG system: EndoG is a nuclease, which naturally resides in the mitochondria due to a presence of a mitochondrial targeting signal (1–48 aa). To re-target EndoG into the nuclear to test its role in oncogenic transformation, the nuclear localization signal (NLS, 5′-GGCCCAAAGAAGAAGAGAAAGGTT-3′ or GPKKKRKV in amino acid sequence) from the SV40 large T antigen was fused to the N-terminal end of a truncated EndoG domain representing amino acid 49–297 (thus missing its native mitochondria targeting signal). The NLS-EndoG cassette was then inserted into the lentivirus vector.

The ORF of human c-Src was amplified from a plasmid pDONR223-SRC (Addgene). PCR products were cloned into the SpeI (New England Biolabs, USA) and NotI (New England Biolabs, USA) sites of the pLEX lentiviral plasmid. The mutations generated were based on extensive literature on SRC structure and function [33]. Constitutively inactive c-Src kinase mutants were generated by PCR amplification using primer pairs Src(F)-Gene/Src(K298M)-R, Src(K298M)-F/Src(Y419F)-R and Src(Y419F)-F/Src(R)-Gene with substituted nucleotides encoding methionine and phenylalanine at residues 298 and 419, respectively. Primer sequences are as follows:

Src-F: 5′-CCGACTCTACTAGAGGATCCACTAGTATGGGTAGCAACAAGAGCAA-3′;

Src-K298M-R: 5′-TTCAGGGTCATGATGGCCACCCTGGTG-3′;

Src-K298M-F: 5′-GTGGCCATCATGACCCTGAAGCCTGGC-3′;

Src-Y419F-R: 5′-CGCGCCGTGAACTCATTGTCTTCAA-3′;

Src-Y419F-F: 5′-GACAATGAGTTCACGGCGCGGCAAG-3′;

Src-R: 5′-CAGGAACATCATACGGATAAGCGGCCGCGAGGTTCTCCCCGGGCTGGT-3′.

### Sorting out caspase-3 reporter-activated cells

To monitor caspase-3 activity during the transformation process, fibroblasts stably expressing the Casp3-Luc-GFP reporter gene were infected with mPOR factor to initiate the transformation process. After 10 days of infection, mPOR-transduced cells were divided into 4 groups (R1–R4) according to their GFP activity using the FACS (BD FACSVantage SE).

### Soft agar colonies formation

About 500–5000 cells inoculated in 6-well plates containing 0.3% Nobel agar. Three weeks after plating, soft agar plates were stained with 0.005% crystal violet. Colonies were then photographed and counted with the Image J software.

### Western blot analysis

Cells were washed 3 times with ice-cold PBS and then cellular lysates were obtained with appropriate volumes of RIPA buffer containing protease inhibitors. Adjustments of 40–60 μg per sample were used for Western blot analysis. Extraction of cytoplasmic and nuclear proteins was performed using a Nuclear and Cytoplasmic Protein Extraction Kit (Beyotime, Shanghai, China). See Supplementary Table 1 for information of antibodies used in western analyses.

### Immunofluorescence staining

Cells were cultured on 4-chamber glass-bottom dishes. After washing three times with PBS, the cells were fixed with 4% paraformaldehyde (PFA) for 15 min, permeabilized and blocked with PBS containing 5% goat serum, 0.1% Triton X-100, and 1% bovine serum albumin (BSA) for 45 min. Blocked cells were incubated with appropriate primary antibodies of cleaved caspase-3, EndoG overnight at 4 °C, and then incubated for 1 h with the appropriate Alexa Fluor 488, 555-conjugated secondary antibodies (Invitrogen, Carlsbad, CA, USA) and mounted with mounting medium (Vector Laboratories, CA, USA) containing DAPI. Fluorescent images were acquired with a Zeiss fluorescence microscope with a 63× oil objective (Axio Observer Z1). The MMTV-PyMT tumor tissues were fixed in 4% paraformaldehyde, dehydrated, embedded in paraffin, and then serially sectioned into 4 μm sections for immunofluorescence staining analysis. Densitometric quantification of fluorescence intensity was performed with Image J software. Confocal pictures were analyzed with two threshold density levels as follows: one for the most fluorescent compartment (nucleus or cytoplasm) and the other for the whole cell. These measurements allowed the calculation of an average density of nuclear or cytoplasmic fluorescence. The integrated density of the nucleus was then divided by the integrated densities of the whole cells to obtain the mean percentage of nuclear EndoG.

### Mouse xenograft tumors

The animal experimental procedures in this study were approved by the Sun Yat-sen University Institutional Animal Use and Care Committee. Six-week-old female BALB/c-nude mice were obtained from Laboratory Animal Center of Sun Yat-sen University, and were randomly divided into groups for cell inoculation. Sample sizes were selected according to commonly accepted standards for intragroup validation of tumor inoculations to achieve statistical significance. The experiment was not conducted in a double-blinded manner. HFF-mPOR, Casp3 KO-mPOR or EndoG KO-mPOR cells (1 × 10^6^) were injected subcutaneously into the flanks of BALB/c-nude mice. Tumor size was measured with a caliper and calculated using the following formula: volume = (length)(width)^2^/2. The endpoint was defined as the time when a progressively growing tumor reached 15 mm in the longest dimension. Tumor samples were collected at the indicated time points and processed for bioanalysis.

### Tissue collection and histological analysis

The xenograft tumors were fixed in 4% paraformaldehyde, dehydrated, embedded in paraffin, and then serially sectioned into 4 μm sections for histopathological analysis. After antigen retrieval, slides were incubated for 15 min in peroxidase blockers (ZSGB-BIO, Beijing, China) to block endogenous peroxidase, and incubated with the corresponding antibodies overnight at 4 °C, and then stained with secondary antibodies for 1 h at room temperature. After staining with DAB (ZSGB-BIO, China), hematoxylin was counterstained. The immunohistochemical staining of sections was quantitatively analyzed by Image J in this study. Average optical density (AOD) was used for statistical analysis. AOD = the integrated optical density (IOD)/Area of positive staining in each IHC staining image.

### Mammary tumorigenesis in MMTV-PyMT transgenic mice

FVB/N-Tg(MMTV-PyMT)634Mul/J (Strain#: 002374, MMTV-PyMT) and B6N.129S1-Casp3 tm1Flv/J (Strain#: 006233, caspase-3 KO) mice from Jackson Laboratory were used in our experiments. MMTV-PyMT male mouse was bred to caspase-3 KO female mouse for F1 generation with a mixed genetic background of B6N.129S1/FVB. Caspase-3 heterozygous/homologous with PyMT genotype mice were then intercrossed to generate PyMT:caspase-3 (WT) and PyMT:caspase-3(KO) mice. The background and generation are shown in Table S2. Mice were housed in pathogen-free environment following the guidelines of Institutional Animal Use and Care Committee of Duke University. The animal experiment was approved by Duke University Institutional Animal Use and Care Committee (IACUC) and conducted according to institutional guidelines. The experiment was not conducted in a double-blinded manner. PyMT:caspase-3 (WT) and PyMT:caspase-3(KO) age-matched female virgin littermates were observed for mammary tumorigenesis experiments. Tumor growth was monitored twice a week by use of a caliper. Tumor-bearing mice were sacrificed once their tumor sizes reached 1.5 cm in diameter. After sacrifice, the final number of tumors were counted for each mouse and total tumor mass for each mouse were also determined.

### Tumor sphere formation

Transformed cells were cultured at low density (1–2 cells/mm^2^) on uncoated plates in tumor sphere growth medium (DMEM/F12 supplemented with nonessential amino acid, glutamine, B-27 supplement without vitamin A, 0.2% heparin, 20 ng/mL EGF, and 25 ng/mL b-FGF). Cells were cultured up to 10 days, during which time they were monitored for tumor sphere formation.

### Limiting dilution assay

Limiting dilution assay for tumor sphere formation assay was performed. Briefly, individual cells were plated in 96-well plates with 0.2 ml/well of sphere growth media DMEM/F12 containing nonessential amino acid, Glutamine, B-27 supplement without vitamin A, 0.2% heparin, 20 ng/ml EGF and 25 ng/ml b-FGF. Limiting dilution method was used to inoculate the cells ranging from 1 to 800 cells/well. Cells were cultured up to 14 days, during which time they were monitored for sphere formation.

### Statistical analysis

All statistical analyses were performed using the GraphPad Prism software (version 9.0, GraphPad Software Inc., San Diego, CA). The data were analyzed using the unpaired Student’s t-test for group comparisons with a normal distribution. The Mann–Whitney *U* test was employed to compare data sets that deviate from a normal distribution within groups. The n number represents n biologically independent experiments in each group. Data are represented as average mean ± SD. In addition, for Kaplan–Meier analysis, the log-rank test was used. In all cases, *p* < 0.05 was defined as statistically significant results.

### Supplementary information

Supporting Information

Original Data

## Data Availability

The data that support the findings of this study are available from the corresponding authors upon reasonable request.
