# Supplementary material for: Caspase-3 promotes oncogene-induced malignant transformation via EndoG-dependent Src-STAT3 phosphorylation
Source: Cell Death Dis. 2024 Jul 9;15(7):486. doi: 10.1038/s41419-024-06884-3 (PMC11231138; doi:10.1038/s41419-024-06884-3)
Supplement: Supplementary file 1 — Supporting Information [file 41419_2024_6884_MOESM1_ESM.pdf]

Supporting Information

Caspase-3 promotes oncogene-induced malignant transformation via EndoG-dependent Src-STAT3 phosphorylation

Chenchen Zhu[1], Fushun Fan[2], Chuan-Yuan Li[3], Yan Xiong[4]*, Xinjian Liu[1]*

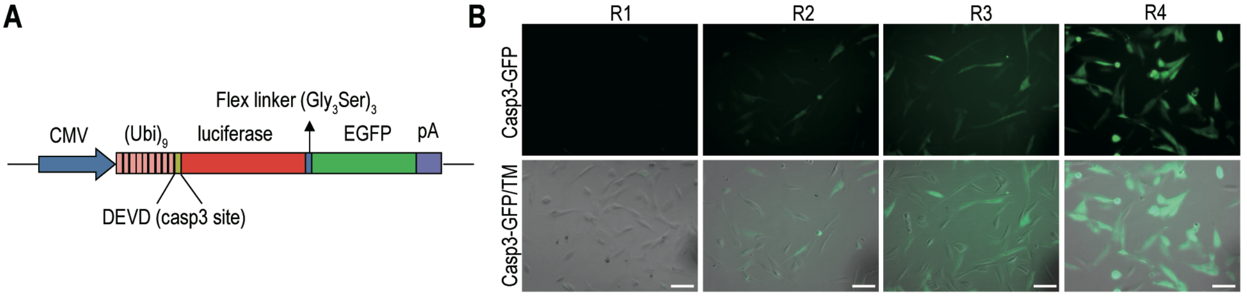

Fig. S1. Caspase-3 activation in oncogene-induced transformation

A, Schematic of caspase-3 reporter that consist of a firefly luciferase-GFP fusion protein (Luc-GFP) linked to a polyubiquitin domain and caspase-3 cleavage site (DEVD).

B, Based the fluorescence intensity of the cells on day 10, mPOR-transduced caspase-3 reporter fibroblasts were sorted into four subpopulations (R1-R4) by use of FACS. Scale bar represents 200 µm.

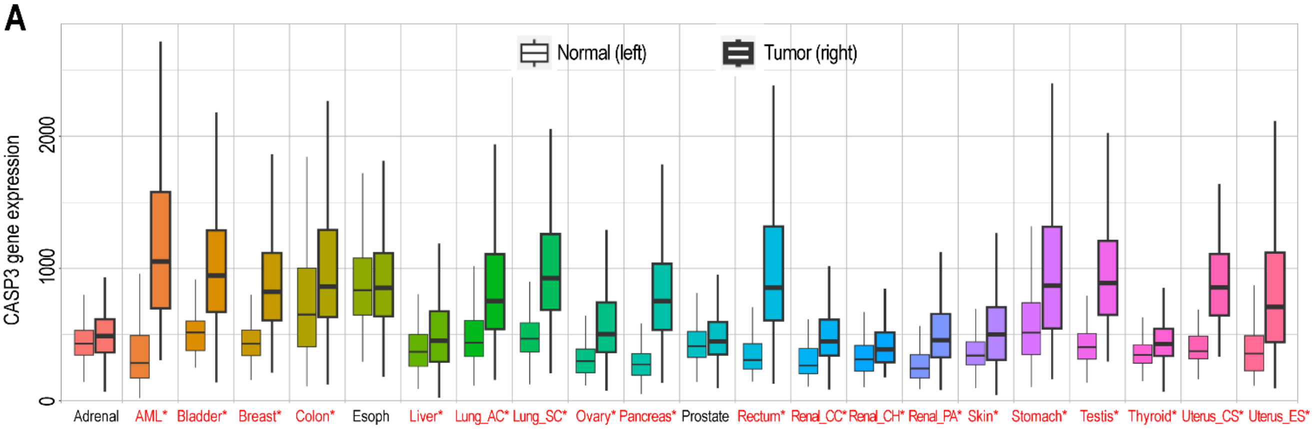

Fig. S2. The expression of caspase-3 is positively correlated with the development of multiple human malignant tumors

A, The expression levels of caspase-3 in different cancer types from TNMplot datasets. Significant differences by Mann-Whitney U test are marked with red*.

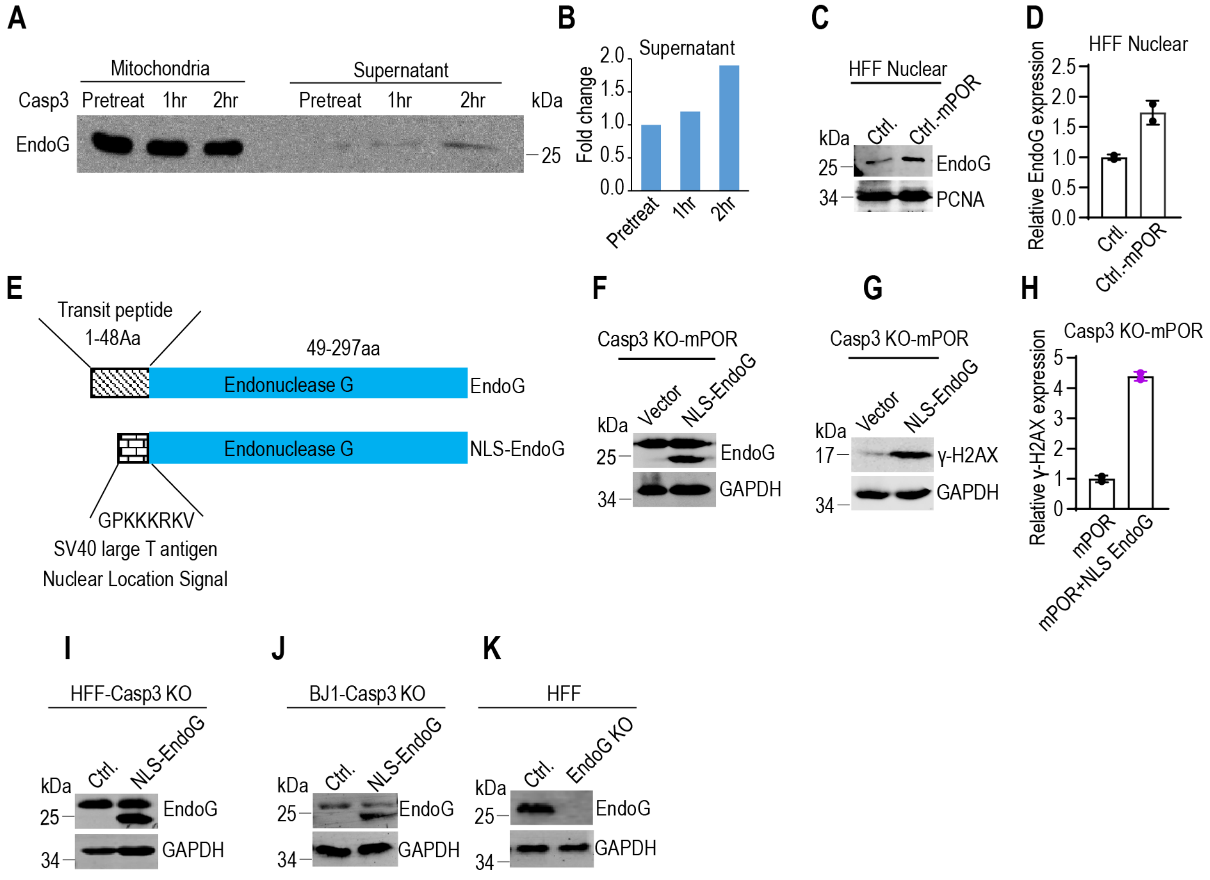

Fig. S3. Endonuclease G as a major downstream effector of caspase-3 mediated transformation

A and **B,** Western blot images (A) and quantification (B) of EndoG expression in mitochondria and supernatant treated with recombinant human caspase-3 protein at different time points. The "Pretreat" indicates the supernatant after 2 hours without the addition of Casp3 in the decontaminant-containing buffer.

C and **D,** Western blot images (C) and quantification (D) of expression of EndoG in the nucleus of mPOR transduced HFF cells. PCNA was used as nuclear protein loading control.

E, A diagram showing a re-engineered EndoG with a nucleus localization signal (NLS) at its tagged N-terminal.

F, Western blot analysis of EndoG expression in Casp3 KO-mPOR-transduced fibroblasts with nuclear-located modified EndoG (NLS-EndoG).

G and **H,** Western blot images (G) and quantification (H) showing γ-H2AX expression in mPOR-transduced Casp3 KO fibroblasts in the presence of NLS-EndoG.

I and **J,** Western blot analysis of EndoG expression in Casp3 KO HFF (I) and BJ1 (J) cells reintroduced with NLS-EndoG.

K, EndoG expression in control (Ctrl.) and EndoG knockout (EndoG KO) HFF cells.

GAPDH was used as protein loading control for (F), (G), (I), (J) and (K).

The blots for the target protein and its corresponding loading control were run on the same gel in each experiment (C,F,G,I,J, and K) , with Fig. S3F, Fig. S3G, Fig. S3I, Fig. S3J, and Fig. S3K being duplicated.

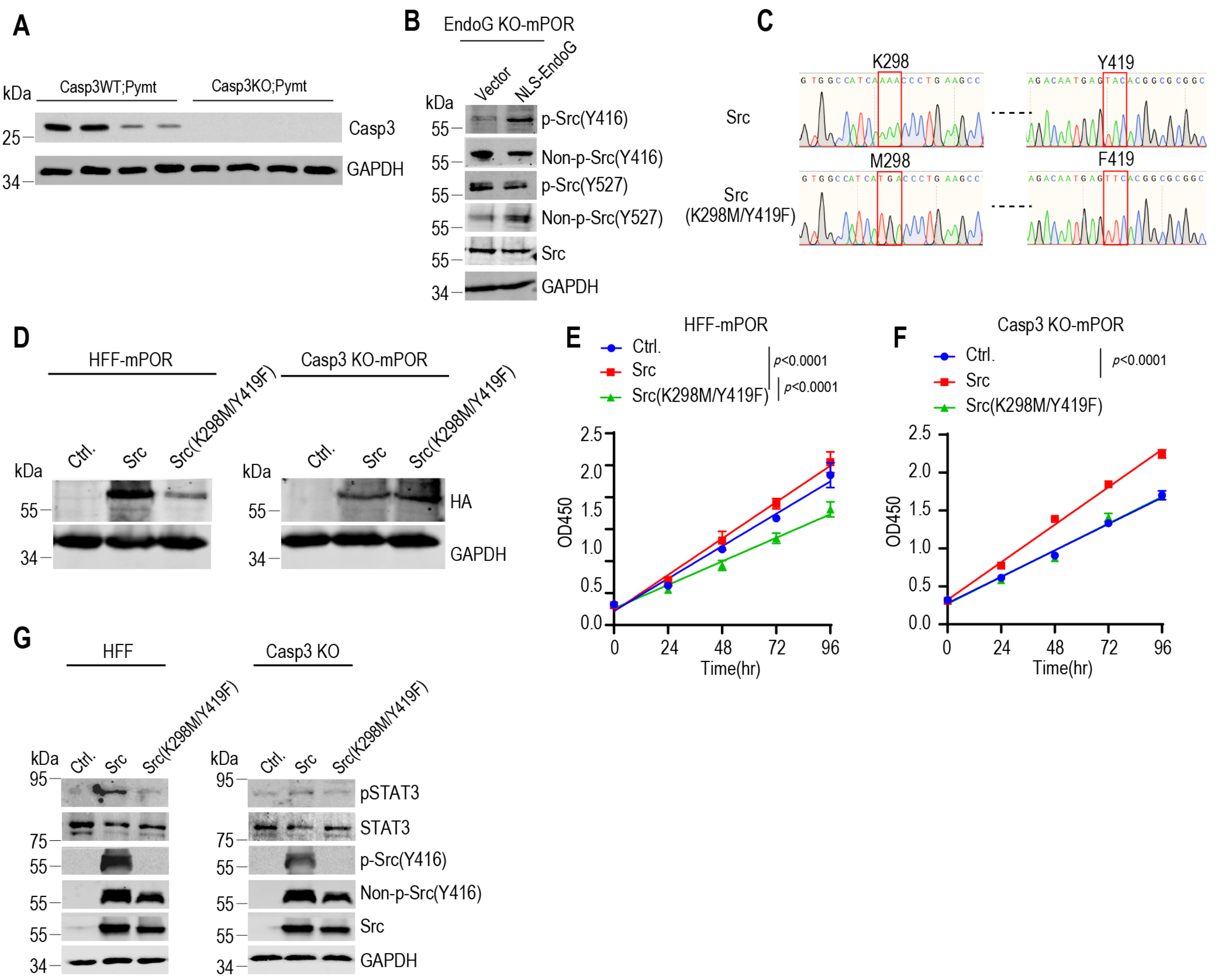

Fig. S4. Src can activate STAT3 signaling pathways

A, Western blot analysis of caspase-3 expression in the tumor tissues from various Casp3WT;Pymt mice and Casp3KO;Pymt mice. GAPDH was used as protein loading control. The samples used for caspase3 and GAPDH blotting were identical to those in Figure 5B.

B, Western blot analysis of phosphorylation and non-phosphorylation of Src at Y416 and Y527 in mPOR-transduced EndoG KO HFF in the presence of NLS-EndoG. GAPDH was used as protein loading control. The blots for the target protein and GAPDH were run on separate gels.

C, Sanger sequencing identified an inactive Src kinase mutant (K298M-Y419F) nucleotide sequence. The red frame indicates the position of the variant.

D, Wild-type Src and an inactivated mutant Src (K298M/Y419F) were introduced into HFF-mPOR and Casp3 KO-mPOR cells through lentiviral infection. Western blotting analysis of the expression level of HA-labeled Src in these cells. GAPDH was used as protein loading control. The blots for the target protein and GAPDH were run on separate gels.

E and **F,** Total viable cells were measured with a CCK-8 assay on the indicated days. n=4. *p* values were determined by two-way ANOVA.

G, Western blot analysis of phosphorylation of Src and STAT3 in HFF and Casp3 KO cells after transfecting with wild-type Src, or inactive Src (K298M-Y419F). GAPDH was used as protein loading control. The blots for the target protein and GAPDH were run on separate gels.

Table S1. Antibodies used in this study

Antibodies	Source	Identifier	Dilution
rabbit anti-caspase3	Cell Signaling Technology	Cat#9665; RRID:AB_2069870	1:1000 for WB
rabbit anti-Cleaved-caspase3	Cell Signaling Technology	Cat#9661; RRID:AB_2341188	1:1000 for WB,1:400 for IF
mouse anti-phospho-Histone H2A.X (Ser139)	Sigma-Aldrich	Cat#05-636	1:1000 for WB
mouse anti-ENDOG (Endonuclease G)	ORIGENE	Cat#TA504189;RRID:AB_11124573	1:1000 for WB,1:100 for IF
pSTAT3	Cell Signaling Technology	Cat#9131; RRID:AB_331586	1:1000 for WB,1:400 for IHC
STAT3	Cell Signaling Technology	Cat#9139; RRID:AB_331757	1:1000 for WB
rabbit anti-Src	Cell Signaling Technology	Cat#2123; RRID:AB_2106047	1:1000 for WB
mouse anti-Non-phospho-Src (Tyr416)	Cell Signaling Technology	Cat#2102; RRID:AB_331358	1:1000 for WB,1:100 for IHC
Phospho-Src Family (Tyr416)	Cell Signaling Technology	Cat#6943; RRID:AB_10013641	1:1000 for WB
rabbit anti-Non-phospho-Src (Tyr527)	Cell Signaling Technology	Cat#2107; RRID:AB_331081	1:1000 for WB
rabbit anti-Phospho-Src (Tyr527)	Cell Signaling Technology	Cat#2105; RRID:AB_331034	1:1000 for WB
mouse anti-GAPDH	Proteintech	Cat#60004-1-Ig; RRID:AB_2107436	1:20000 for WB
mouse anti-PCNA	Proteintech	Cat#60097-1-Ig; RRID:AB_2236728	1:5000 for WB
rabbit anti-HA	Cell Signaling Technology	Cat#3724	1:1000 for WB
mouse anti-β-Actin	Proteintech	Cat#66009-1-Ig; RRID:AB_2687938	1:20000 for WB

rn blot, IF: Immunofluorescence, IHC: Immunohistochemistry

Table S2. Phenotypic information of 156 mice.

Genotype	Gender	Number	Number of mice in each generation
$Casp3^{+/-}$(without Pymt)	Female	9	F1(3), F2(2), F3(4)
	Male	6	F1(1), F2(4), F3(1)
$Casp3^{+/-}$;Pymt	Female	19	F2(5), F3(14)
	Male	13	F1(1), F2(2), F3(10)
$Casp3^{+/+}$(without Pymt)	Female	12	F1(3), F2(4), F3(5)
	Male	8	F2(5), F3(3)
$Casp3^{+/+}$;Pymt	Female	18	F2(7), F3(11)
	Male	15	F1(1), F2(2), F3(12)
$Casp3^{-/-}$(without Pymt)	Female	16	F2(7), F3(9)
	Male	9	F2(3), F3(6)
$Casp3^{-/-}$;Pymt	Female	20	F3(20)
	Male	11	F3(11)
