# Supplementary material for: Caspase-3 promotes oncogene-induced malignant transformation via EndoG-dependent Src-STAT3 phosphorylation
Source: Cell Death Dis. 2024 Jul 9;15(7):486. doi: 10.1038/s41419-024-06884-3 (PMC11231138; doi:10.1038/s41419-024-06884-3)

Fig. 1C

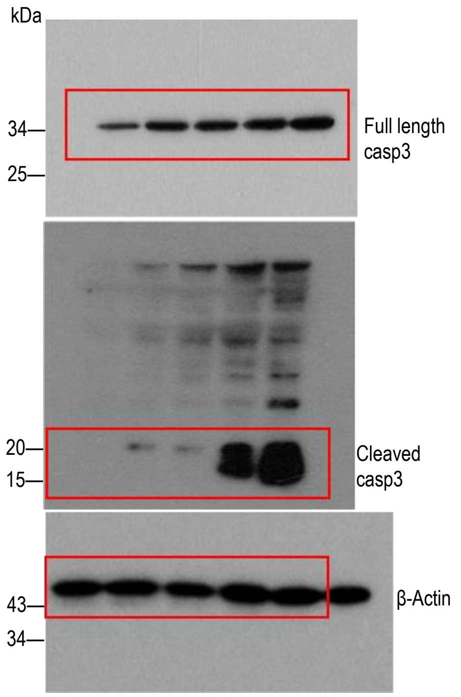

Fig. 2A

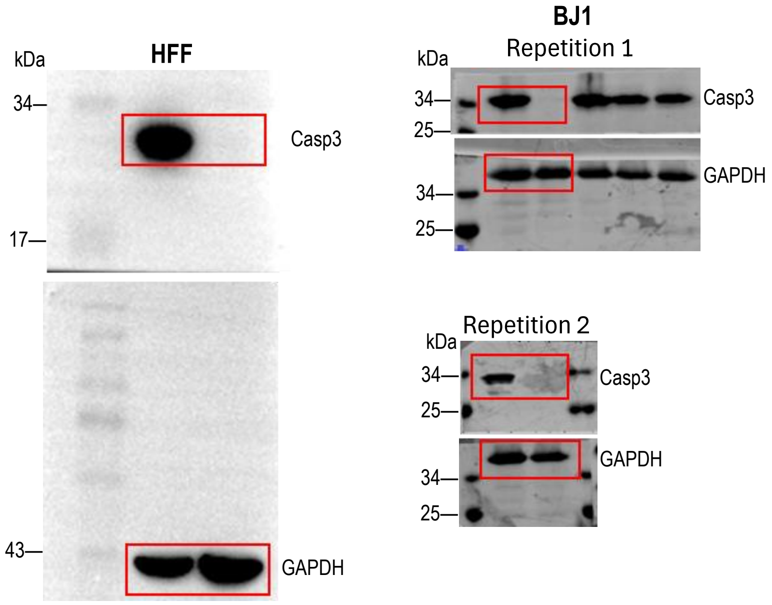

Fig. 4C

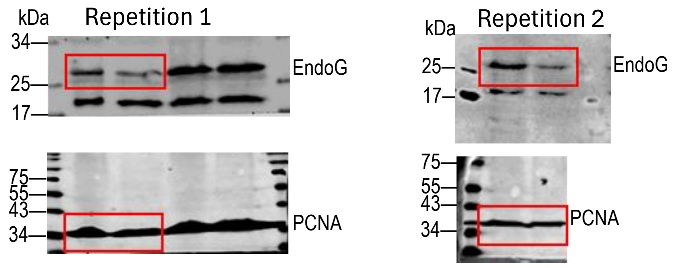

Fig 4G

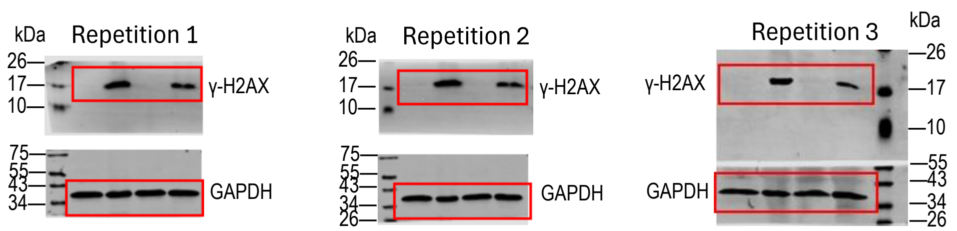

Fig. 5A

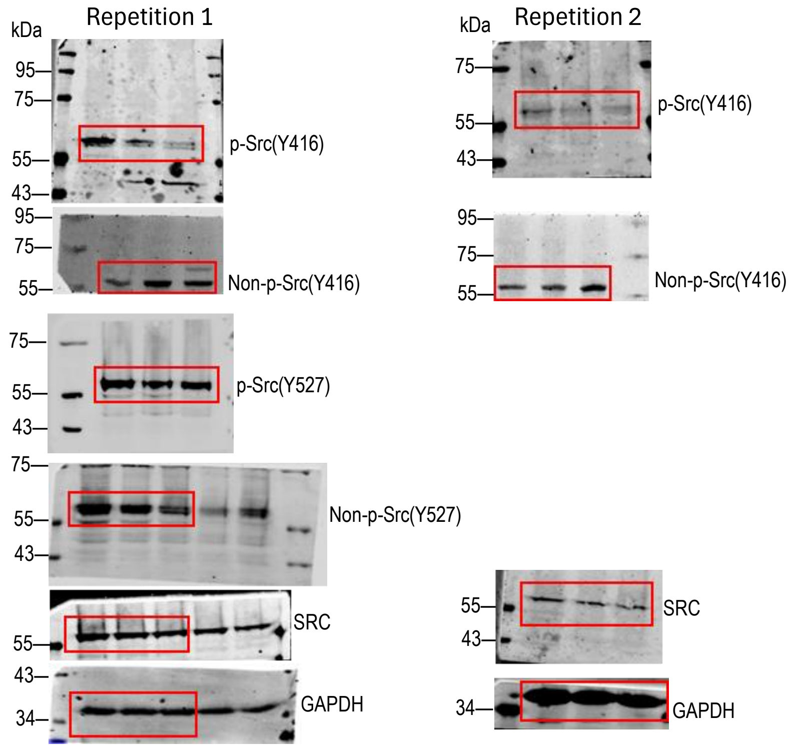

Fig. 5B

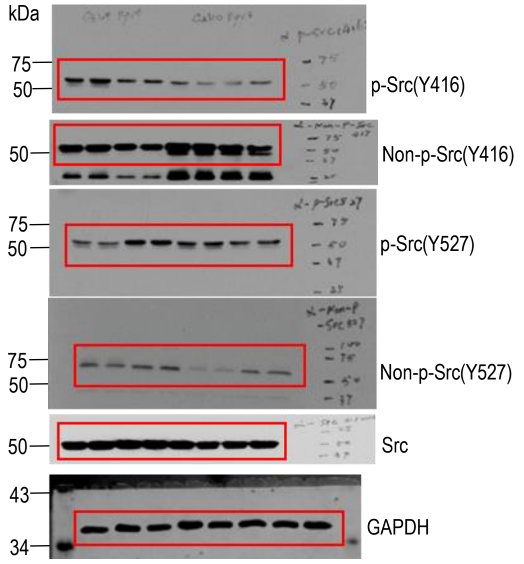

Fig. 5C

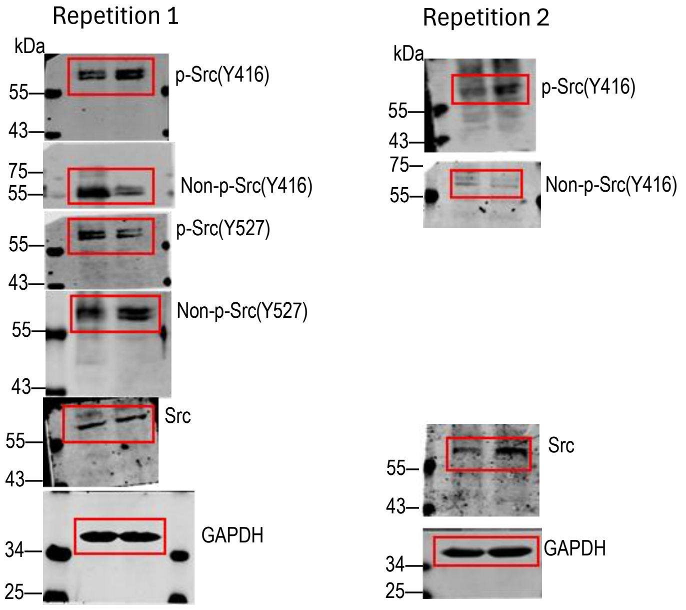

Fig. 5F

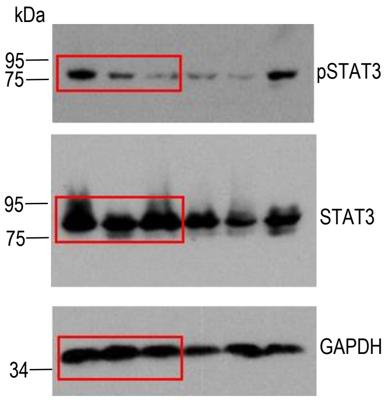

Fig. 5G

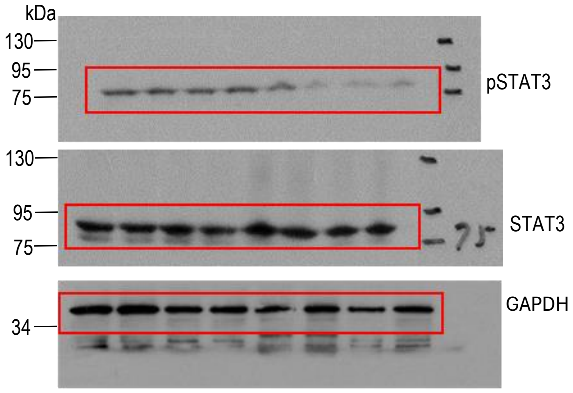

Fig. 5H

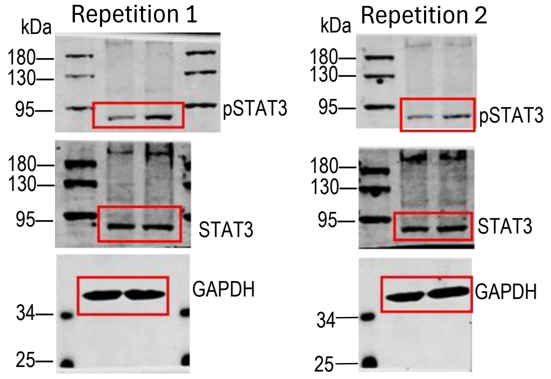

Fig. S3A

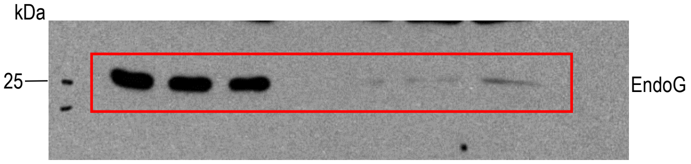

Fig. S3C

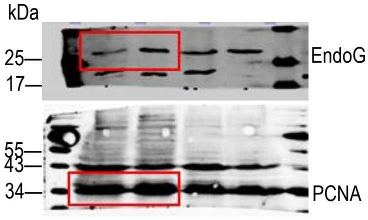

Fig. S3F

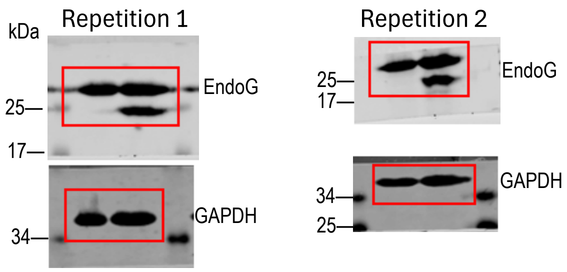

Fig. S3G

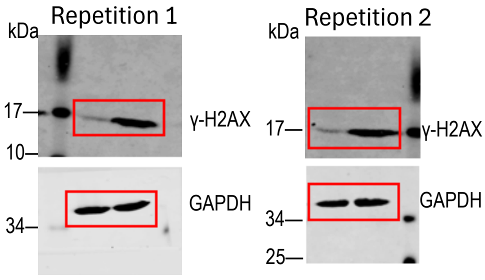

Fig. S3I

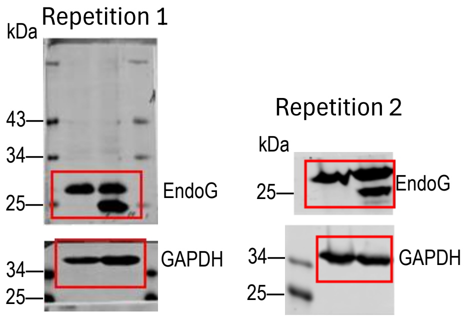

Fig. S3J

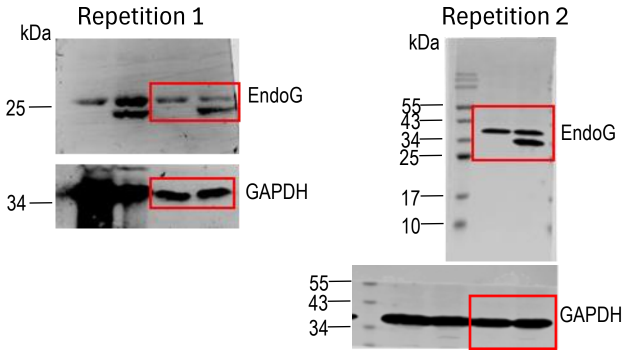

Fig. S3K

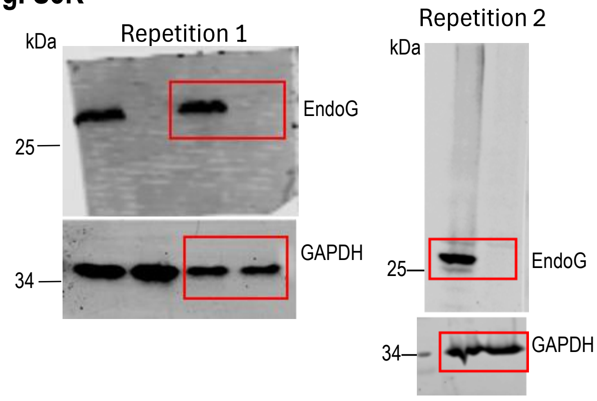

Fig. S4A

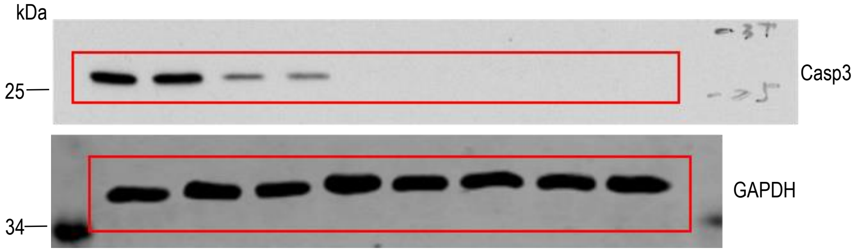

Fig. S4B

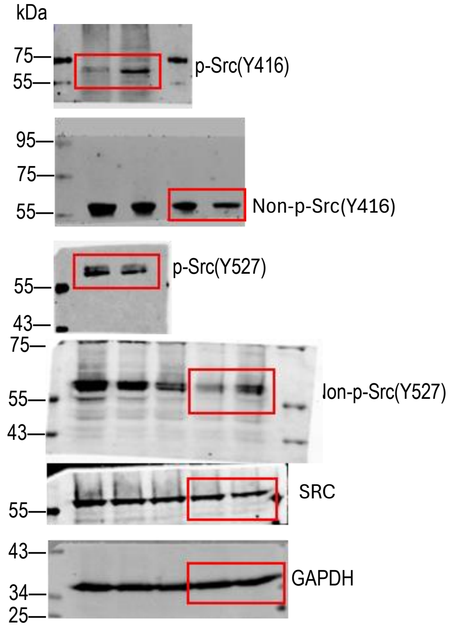

Fig. S4D

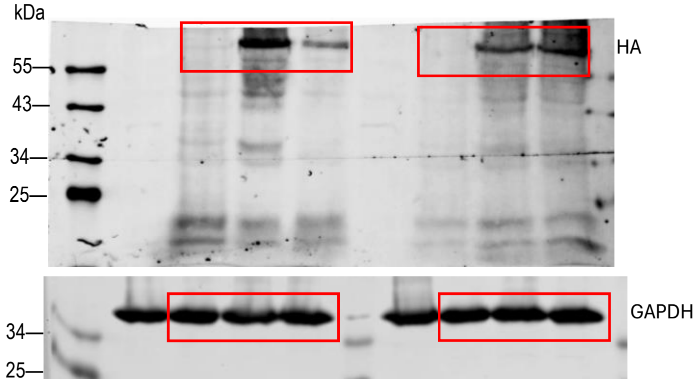

Fig. S4G

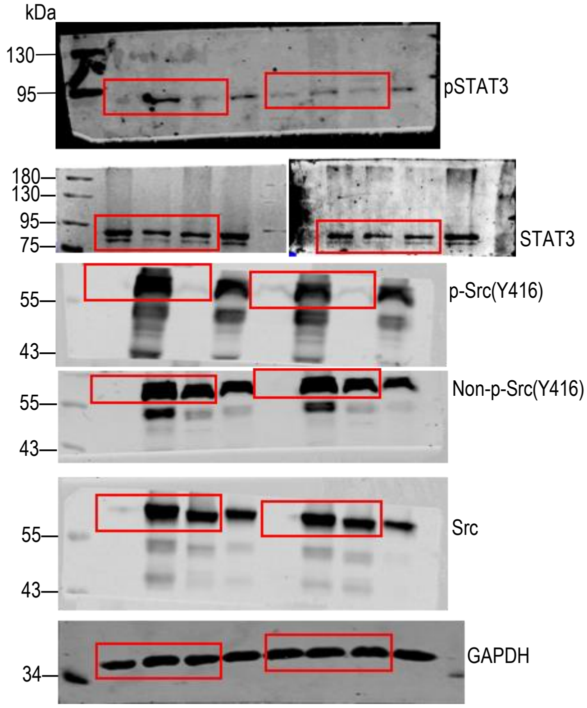

Supplement: Supplementary file 2 — Original Data [file 41419_2024_6884_MOESM2_ESM.pdf]
